# Real-world evidence on childhood refractive errors in a developed coastal region of China: a multi-center analysis of 4.47 million school-based screening encounters in Southern Fujian

**DOI:** 10.3389/fpubh.2026.1938109

**Published:** 2026-08-24

**Authors:** Xiao-feng Li, Dong-kan Li, Wei Liang, Peng Shi, Bin Lin

**Affiliations:** 1 Xiamen Eye Center and Eye Institute of Xiamen University, School of Medicine, Xiamen, China; 2 Xiamen Clinical Research Center for Eye Diseases, Xiamen, Fujian, China; 3 Xiamen Key Laboratory of Ophthalmology, Xiamen, Fujian, China; 4 Fujian Key Laboratory of Corneal & Ocular Surface Diseases, Xiamen, Fujian, China; 5 Xiamen Key Laboratory of Corneal & Ocular Surface Diseases, Xiamen, Fujian, China; 6 Translational Medicine Institute of Xiamen Eye Center of Xiamen University, Xiamen, Fujian, China

**Keywords:** health policy, myopia, real-world evidence, school-based screening, uncorrected refractive error

## Abstract

**Background:**

Despite rapid economic development in coastal China, the alignment between socioeconomic growth and pediatric eye health remains poorly understood. School-based screening may provide an opportunity to bridge this knowledge gap, yet large-scale, real-world evidence from diverse urban-rural gradients is scarce.

**Methods:**

This cross-sectional study analyzed 4.47 million valid screening encounters from Xiamen, Quanzhou, and Zhangzhou (2021–2024). Visual acuity (VA) and spherical equivalent (SE) were assessed. Two-way ANOVA and multivariate logistic regression were employed to identify drivers of visual impairment and uncorrected refractive error, with screening year as a covariate. Generalized Estimating Equations (GEE) with student-level clustering were applied to account for repeated measures. Sensitivity analyses confirmed dataset robustness.

**Results:**

Age was the predominant driver of VA variance (η^2^ = 0.1132), exceeding city-level effects fivefold. Xiamen exhibited the best refractive profile but the highest uncorrected rate (94.0% in primary school), illustrating the “Xiamen Paradox”—where affluence fosters a “visibility-over-correction” bias. Conversely, Quanzhou demonstrated a “pain-driven” model with high correction adherence despite a severe myopia burden (19.0% high myopia). Crucially, county-level SE disparities (up to 1.66 D) eclipsed inter-city gaps. The 5–7-year window marked a critical refractive shift, with myopia prevalence doubling. Among primary school records, only 22.6% of visually impaired children wore spectacles, and merely half achieved adequate correction.

**Conclusion:**

Economic prosperity does not automatically translate to optimal visual health. Significant “correction gaps” and intra-regional disparities necessitate a shift from uniform strategies to precision governance. Future interventions must prioritize the 5–7-year window, emphasize correction quality over mere screening volume, and tailor approaches to local socioeconomic contexts to transform economic capital into sustainable ocular health dividends effectively.

## 1 Background

School-based vision screening holds substantial public health significance in China. Extensive studies indicate a growing burden of visual impairment among children and adolescents, particularly due to the rising prevalence of myopia. Early-onset myopia is prone to progression toward high myopia, thereby increasing the risk of irreversible blindness (1, 2). Since over 90% of visual impairment is preventable, yet routine eye screening remains unestablished in primary healthcare systems, schools serve as critical venues for large-scale vision intervention (3). Such screening programs facilitate the early detection of common conditions, including refractive errors and amblyopia, while providing essential epidemiological evidence to inform targeted prevention strategies (4). Research demonstrates that screening enables the identification of distinct visual trajectory subgroups, supporting stratified management approaches.

Furthermore, school-based initiatives significantly improve access to eye care services for vulnerable populations—such as children of rural residents and migrant workers—thereby mitigating treatment disparities driven by socioeconomic inequalities (5–7). Notably, uncorrected refractive error impairs not only visual function but also academic performance and psychological well-being; conversely, school-based spectacle interventions have been proven to enhance educational outcomes (8, 9). The integration of digital technologies or artificial intelligence into screening protocols offers superior cost-effectiveness compared to traditional methodologies, particularly in resource-limited settings (10, 11). Concurrently, school environmental factors—including green space coverage and classroom lighting—critically influence visual development; thus, screening data provide actionable insights for optimizing the school visual environment (12–14). Accordingly, promoting standardized, routine school-based vision screening constitutes a cornerstone strategy for actualizing China's National Myopia Prevention and Control Plan and advancing universal eye health objectives.

As a pivotal economic hub along China's southeastern coast, Fujian Province serves not only as a frontline zone for cross-strait integration and development but also as a bellwether for national economic progress. The Southern Fujian Golden Triangle—comprising Xiamen, Quanzhou, and Zhangzhou—functions as the province's economic engine, contributing nearly 50% of the provincial Gross Domestic Product (GDP) in 2024; consequently, its socio-developmental paradigm offers significant referent value for China's vast coastal developed regions. Notably, while China has transitioned into a middle-high income country, the three cities within Southern Fujian exhibit a research-worthy “gradient development” characteristic: Xiamen, a Special Economic Zone, boasts a per capita GDP surpassing thresholds typical of developed nations and possesses superior medical and educational resources; Quanzhou demonstrates robust private sector dynamism with a trillion-yuan GDP, yet its substantial population of migrant workers' children presents unique challenges to pediatric eye health; Zhangzhou, though comparatively smaller in economic aggregate, displays marked intra-regional heterogeneity, encompassing highly urbanized districts alongside relatively underdeveloped agricultural counties, thereby providing a natural experimental field to explore refractive error governance across varying socio-economic development stages. The pronounced disparities among these cities—spanning economic scale, demographic composition, and healthcare resource allocation—make local vision health data representative of both the shared attributes of developed coastal areas and the distinct characteristics of varied developmental phases, thus holding considerable potential to inform national “economy-health” co-development models.

Nevertheless, economic prosperity does not automatically translate into commensurate improvements in population vision health. In Southern Fujian, rapid urbanization, intense academic pressures, and highly mobile demographic structures may constitute distinct risk factors for visual impairment. In particular, asymmetries in developmental models, fiscal investment intensities, and primary eye care capacities among the three municipalities could lead to significant disparities in the effectiveness of myopia control. Currently, systematic reports that leverage large-scale, longitudinal, standardized screening data from this region remain scarce; notably absent are comparative studies grounded in real-world evidence that elucidate vision health governance models across economic gradients. Accordingly, conducting comprehensive, multi-city school-based vision screenings across Southern Fujian represents not only a proactive response to the national myopia prevention strategy but also a critical endeavor to delineate the baseline burden and disentangle the complex nexus between “economic growth and health outcomes.” Employing a robust, large-sample dataset, this study aims to characterize the current landscape and systemic gaps in regional vision health, thereby furnishing pivotal evidence to address latent governance challenges—such as the “high-growth, low-correction” paradox—and to underpin the construction of a precision myopia prevention framework aligned with China's specific national context.

## 2 Methods

### 2.1 Study design and data source

This study was a large-scale, regional cross-sectional survey. Data were derived from student vision screening programs conducted in Xiamen, Quanzhou, and Zhangzhou, Fujian Province, spanning 2019–2024. Preliminary data inspection revealed considerable missing items—including key refractive and visual acuity (VA) parameters—during the initial phase of the COVID-19 pandemic (2019–2020), attributable to disrupted school schedules and inconsistent screening protocols. To ensure analytical validity and minimize potential confounding, these records were excluded from the primary analysis. Consequently, the final analyzed dataset comprised 4,475,132 valid screening encounters from 2021 to 2024.

Between 2021 and 2024, a total of 4,475,132 screening encounters were recorded from 1,623,070 unique students across 1,361 schools in Xiamen, Quanzhou, and Zhangzhou. On average, each student contributed 2.76 screening records (median = 3, interquartile range: 1–4). To address potential bias from repeated measurements, all prevalence estimates are reported at the encounter level to reflect real-world screening utilization. At the same time, sensitivity analyses were performed at the student level (using the most recent valid measurement per student) and through Generalized Estimating Equations (GEE) with clustering on student ID.

### 2.2 Data cleaning and quality control

The data cleaning protocol was implemented as follows: Of the initial 5,270,848 records, 489,230 entries with anomalous age data were excluded (including age = 0, failed cross-validation, age < 3 years, and age > 19 years). Subsequently, 670 records were removed due to invalid VA measurements (e.g., non-cooperation resulting in zero acuity, or non-numeric text entries such as “no light perception,” “hand motion,” or “finger counting”). Additionally, 25,460 records lacking information on the correction method but possessing valid corrected VA data were logically recoded as “spectacle-wearers (method unknown).” Sensitivity analyses indicated that the inclusion or exclusion of 2019–2020 data exerted negligible effects on core metrics (mean spherical equivalent (SE) and myopia prevalence), with differences in SE remaining within the measurement error margin (< 0.04 D). This confirms the robustness of the 2021–2024 dataset.

### 2.3 Variable definitions and measurements

#### 2.3.1 Visual acuity (VA)

Consistent with current Chinese screening practices, unaided (uncorrected) VA and aided (corrected) VA were measured using the standard logarithmic visual acuity chart.

#### 2.3.2 Refractive parameters

SE was obtained via non-cycloplegic automated refractometry using the Topcon KR-8900 autorefractor (Topcon Corporation, Tokyo, Japan). All devices underwent routine annual calibration according to manufacturer specifications. SE was calculated as: SE = Sphere + 1/2 Cylinder. Myopia was defined as SE ≤ −0.50 D. Given the large-scale nature of school-based screening, cycloplegia was not administered.

#### 2.3.3 Correction status

Two complementary metrics were used. First, uncorrected visual impairment was defined as presenting VA < 5.0 in the absence of spectacles at screening. Second, uncorrected myopia was defined as SE ≤ –0.5D without spectacles, representing the core public health indicator. To minimize potential misclassification, students were classified as spectacle wearers not only when spectacles were explicitly documented but also when a valid corrected VA was measured despite an undocumented correction status. Internal validation indicated that corrected VA was likewise missing in 99.2% of records with undocumented correction status, supporting the robustness of this conservative classification approach. Adequate correction was defined as achieving corrected VA ≥ 5.0.

#### 2.3.4 Age stratification

Based on chronological age, participants were categorized into four groups: preschool ( ≤ 6 years), primary school (7–12 years), junior high school (13–15 years), and senior high school (≥16 years).

### 2.4 Statistical analysis

Given the high linear correlation between ocular measurements of both eyes (*r* > 0.9), statistical analyses primarily used data from the right eye. Continuous variables were expressed as mean ± standard deviation (x̄±s). Inter-group comparisons were performed using Two-way Analysis of Variance (ANOVA), with the screening year incorporated as a covariate to adjust for temporal trends. Categorical variables were presented as frequencies and percentages (%), with comparisons evaluated using the Chi-square (χ^2^) test. Correlation analyses were conducted using Pearson's correlation coefficient. To quantify the effect size for categorical variables, Cramér's V was calculated, with values of 0.1–0.3 indicating a weak effect and 0.3–0.5 indicating a medium effect.

Logistic regression models were employed to analyze risk factors for visual impairment and uncorrected refractive error. Multivariate models identified predictors of visual impairment (Model 1) and, among those impaired, the likelihood of remaining uncorrected (Model 2). All models generated odds ratios (ORs) with 95% confidence intervals (CIs). Given the massive sample size, we prioritized presenting ORs and effect sizes in the main text. Given the hierarchical data structure (repeated measures within students, students nested within schools and counties), we refitted key models using GEE with an exchangeable correlation structure, clustering on student ID, to obtain cluster-robust standard errors. The intraclass correlation coefficient (ICC) for repeated measures within students was 0.473, confirming substantial within-individual correlation. Sensitivity analyses were additionally performed using one record per student (most recent measurement) and with school-level clustering. While fully specified Bayesian multilevel logistic regression (BMMLR) offers theoretical advantages for jointly modeling multiple binary outcomes (e.g., visual impairment, uncorrected status, adequate correction) within a unified framework, its application was computationally prohibitive at this scale (>4 million encounters, >1.6 million students). Effect sizes were emphasized alongside *p*-values, given the large sample size.

All tests were two-tailed. Given the large sample size, effect sizes were emphasized alongside *P*-values to evaluate clinical significance. All statistical procedures were performed using SPSS software version 22.0 (IBM Corp., Armonk, NY, USA). A detailed STROBE-compliant participant flow diagram is provided in Supplementary Figure S1.

## 3 Results

### 3.1 Baseline characteristics of the study population

A total of 4,475,132 valid screening records were ultimately included in the analysis. The distribution by city was as follows: Xiamen, 1,948,385 records (43.5%); Zhangzhou, 1,865,845 records (41.7%); and Quanzhou, 660,902 records (14.8%). Regarding the age structure, the primary school group accounted for the largest share (50.0%), followed by the junior high school group (27.4%) and the senior high school group (15.3%). Detailed demographic information is provided in Tables 1, 2. Notably, Zhangzhou had a significantly higher proportion of senior high school students (22.1%) than Xiamen (9.6%), indicating heterogeneity in the age distribution of screened populations across the three cities and necessitating stratified analyses. With respect to gender composition, males accounted for 53.9% of records, females for 45.7%, and gender was unspecified in 0.4%.

**Table 1:** Distribution of valid screening records by city and age group.

City	Preschool ( ≤ 6y)	Primary (7-12y)	Junior high (13-15y)	Senior high (≥16y)	Total
Xiamen	182,122 (9.3%)	1,109,337 (56.9%)	470,504 (24.1%)	186,422 (9.6%)	1,948,385
Quanzhou	68,111 (10.3%)	344,063 (52.1%)	161,882 (24.5%)	86,846 (13.1%)	660,902
Zhangzhou	76,368 (4.1%)	782,213 (41.9%)	594,756 (31.9%)	412,508 (22.1%)	1,865,845
**Total**	**326,601**	**2,235,613**	**1,227,142**	**685,776**	**4,475,132**

**Table 2:** Proportional distribution of screening records by city and age group.

City	Preschool	Primary	Junior high	Senior high	Total
Xiamen	9.3%	56.9%	24.1%	9.6%	100%
Quanzhou	10.3%	52.1%	24.5%	13.1%	100%
Zhangzhou	4.1%	41.9%	31.9%	22.1%	100%

### 3.2 Association between uncorrected VA, age, and city

Two-way ANOVA revealed that age group was the predominant driver of variance in uncorrected VA, accounting for 11.32% of the variance (η^2^ = 0.1132), representing an effect size approximately five times greater than that of city (η^2^ = 0.0211). The residual term—representing unmeasured individual factors such as genetics and behavioral habits—accounted for 85.5% of the total variance.

Uncorrected VA declined progressively with age, decreasing from a mean of 4.88 in the preschool group to 4.48 in the senior high school group. *Post hoc* Tukey HSD tests confirmed statistically significant differences in VA among the three cities (all *p* < 0.001). Analysis of gender differences indicated no significant disparity during the preschool period; however, starting from primary school, female students exhibited significantly poorer VA than males, with this gap widening progressively with age (female-male mean difference: −0.053 in the senior high school group).

### 3.3 Distribution of refractive status (SE)

Refractive data revealed more pronounced inter-city disparities. During the preschool period, children in Xiamen exhibited physiological hyperopia (+0.31 D). In contrast, those in Zhangzhou were already in a myopic state (−0.97 D), suggesting that regional differences in refractive status may originate in the pre-school years (see Table 3 for details).

**Table 3:** Mean SE and myopia prevalence by city and age group.

Age group	Xiamen SE	Xiamen myopia rate	Quanzhou SE	Quanzhou myopia rate	Zhangzhou SE	Zhangzhou myopia rate
Preschool ( ≤ 6y)	+0.31D	6.6%	−0.07D	23.9%	−0.97D	39.2%
Primary (7–12y)	−0.75D	40.9%	−1.28D	60.0%	−1.17D	53.6%
Junior high (13–15y)	−2.38D	77.4%	−3.04D	87.3%	−2.42D	84.0%
Senior high (≥16y)	−3.10D	85.9%	−3.75D	91.3%	−2.85D	87.5%

By the primary school stage, the mean SE values were −0.75 D in Xiamen, −1.28 D in Quanzhou, and −1.17 D in Zhangzhou. Two-way ANOVA demonstrated that the effect size of age on SE (η^2^ = 0.1586) vastly exceeded that of city (η^2^ = 0.0077). The prevalence of high myopia showed a particularly polarized distribution: the rate in Quanzhou's senior high school group reached 19.0%, more than double that of Xiamen (8.6%; Table 4 for details).

**Table 4:** High myopia prevalence by city and age group.

Age group	Xiamen	Quanzhou	Zhangzhou
Preschool ( ≤ 6y)	0.1%	0.4%	1.0%
Primary (7–12y)	0.6%	2.3%	1.2%
Junior high (13–15y)	4.3%	11.7%	4.1%
Senior high (≥16y)	8.6%	19.0%	7.0%

Sensitivity analyses revealed that after controlling for SE stratification, inter-city differences in uncorrected VA narrowed substantially. Among individuals with borderline refractive status (−0.5 to 0 D), VA was nearly identical across the three cities (difference = 0.014, < 0.05). Similarly, in the emmetropic (0 to +0.5 D) and myopic (−3 to −1 D) strata, mean VA differences were 0.055 and 0.068, respectively—both below the 0.10 threshold commonly applied in international clinical research (15, 16). These findings suggest that observed inter-city VA disparities are unlikely to stem from measurement bias.

### 3.4 Prevalence of visual impairment and uncorrected refractive error

Although Xiamen had the lowest prevalence of visual impairment among students (46.4% in the primary school group), it exhibited the highest uncorrected rate at 94.0% (primary school group), ranking first among the three cities. Conversely, Quanzhou reported the highest prevalence of visual impairment (57.9% in the primary school group); however, benefiting from the subjective urgency associated with blurred vision, its uncorrected rate was considerably lower at 61.4% (primary school group; Table 5). Crucially, these raw prevalence disparities persist after adjusting for age and sex distributions: multivariate logistic regression demonstrated that, relative to Zhangzhou, Xiamen students had 60% lower odds of visual impairment (OR = 0.40, *p* < 0.001) but 445% higher odds of remaining uncorrected once impaired (OR = 5.45, *p* < 0.001). In contrast, Quanzhou students exhibited 46% lower odds of visual impairment (OR = 0.54, *p* < 0.001) coupled with 81% lower odds of being uncorrected (OR = 0.19, *p* < 0.001), corroborating the “Xiamen Paradox” and “Quanzhou Model” beyond crude rate comparisons.

**Table 5:** Prevalence of VI and uncorrected refractive error by city and age group (%).

Age group	Xiamen VI rate	Xiamen uncorrected rate	Quanzhou VI rate	Quanzhou uncorrected rate	Zhangzhou VI rate	Zhangzhou uncorrected rate
Preschool ( ≤ 6y)	30.0	97.9	53.3	91.7	77.4	87.2
Primary (7–12y)	46.4	94.0	57.9	61.4	71.6	82.1
Junior high (13–15y)	76.0	91.1	81.0	33.9	84.2	65.3
Senior high (≥16y)	83.7	91.4	86.7	25.6	85.8	56.2

It is necessary to emphasize that the denominator for the uncorrected rate comprised all records with a presenting VA < 5.0. Students were classified as spectacle-wearers if they were documented as wearing spectacles at screening or if a valid corrected VA was measured despite an unrecorded correction type. This conservative approach minimized potential misclassification of correction status.

χ^2^ tests revealed a progressive, age-dependent widening of inter-city disparities in uncorrected refractive error rates. The Cramér's V value increased from 0.16 (weak association) in the preschool group to 0.42 (moderate association) in the senior high school group, indicating that age acts as a key effect modifier amplifying regional differences. Multivariate logistic regression analysis, adjusted for age and gender, further substantiated these findings, confirming the persistence of the “Xiamen Paradox” and “Quanzhou Model” independent of demographic composition. The stability of these effect sizes across model specifications underscores that age acts as a key effect modifier, amplifying regional disparities in uncorrected refractive error rates as students progress through senior high school.

Sensitivity analyses confirmed that the choice of analytic unit did not alter the substantive conclusions. Restricting analyses to one record per student yielded prevalence and OR estimates within 2% points of encounter-level results. Furthermore, GEE models with student-level clustering produced nearly identical effect sizes (e.g., Xiamen vs. Zhangzhou OR for visual impairment: 0.35–0.40; OR for remaining uncorrected: 4.47–5.45 across model specifications). These findings indicate that the “Xiamen Paradox” and “Quanzhou Model” are robust to analytic unit selection and clustering adjustments. These multi-pronged sensitivity approaches collectively support the validity of inferences drawn from our clustered data structure.

### 3.5 Correction modalities and optical quality

Spectacle wear constituted the predominant form of refractive correction (91.7% of corrected individuals). However, significant geographical disparities in orthokeratology (OK) lens penetration were evident: the overall utilization rate in Xiamen was 1.68%, compared to a mere 0.08% in Zhangzhou—a 21-fold difference.

Regarding correction quality, wearing spectacles improved mean VA by 0.62 (approximately three lines on the logMAR chart). Nevertheless, the overall rate of adequate correction was only 50.2%. Significant inter-city differences were observed: Xiamen (60.9%) > Zhangzhou (52.7%) > Quanzhou (41.6%). This indicates that nearly half of the students who had acquired spectacles failed to achieve optimal optical correction, highlighting notable deficiencies in refractive accuracy and prescription updating within Quanzhou.

Notably, adequate correction rates were highly consistent across two analytical cascades: 50.2% among all screened students with corrected VA measured and 48.8% among visually impaired students documented as spectacle-wearers, indicating minimal bias from differential availability of corrected VA data.

### 3.6 County-level heterogeneity and critical intervention windows

Analysis at the county level uncovered even more striking disparities than those observed between cities. Within Zhangzhou municipality, the difference in mean SE between the least affected county (Zhangpu, SE = −0.62 D) and the most severely affected county (Hua‘an, SE = −2.28 D) reached 1.66 D—far exceeding the maximum inter-city difference (~0.5 D). Notably, Hua'an county exhibited paradoxical findings, with an extremely myopic SE (−2.28 D) juxtaposed with a low reported myopia prevalence (26.7%), which may suggest potential localized issues warranting a data quality reassessment.

**Figure 1:**
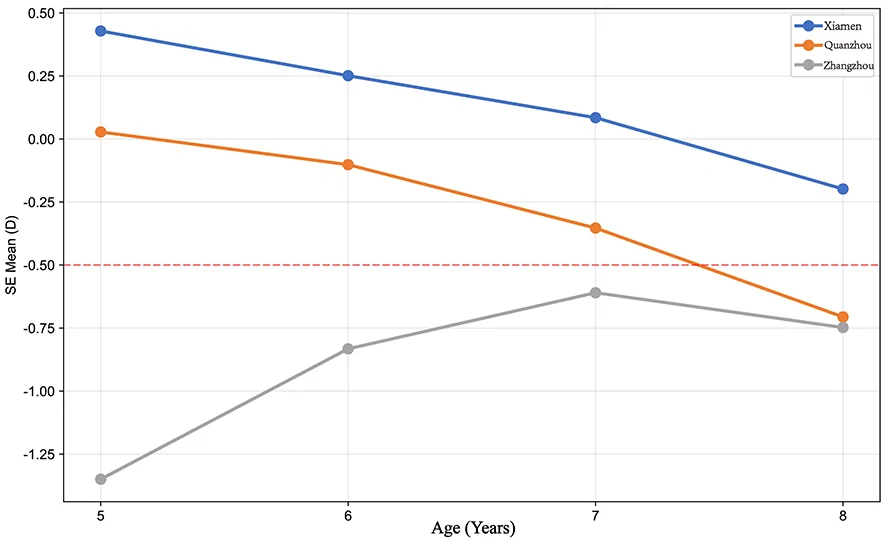
Trajectory of SE during the preschool-to-primary school transition (ages 5–8 years). The figure illustrates a critical refractive shift, highlighting distinct differences in ocular refractive status before and immediately following primary school entry.

Temporal analysis of age-related trends identified a critical intervention window: between the ages of 5 and 7 years, the mean SE decreased by 0.22 D, coinciding with a doubling of myopia prevalence (13.5% → 21.6%). This marks an abrupt transition from the consumption of hyperopic reserves to the onset of myopia (Table 6 and Figure 1). Furthermore, the prevalence of anisometropia (interocular SE difference ≥ 1.0 D) increased dramatically with age, rising from 6.8% in the preschool group to 32.1% in the senior high school group, thereby elevating the risk of amblyopia.

**Table 6:** Changes in refractive status among records aged 5–8 years.

Age (years)	Screening encounters	Mean spherical equivalent (D)	Myopia prevalence (%)
5	43,809	+0.10	13.5
6	185,313	+0.03	15.8
7	308,548	−0.12	21.6
8	305,025	−0.41	32.5

## 4 Discussion

Drawing on the largest single student vision-screening database in China's economically dynamic southeast coastal region, this study presents a comprehensive panorama of pediatric ocular health across three cities in Southern Fujian. Our findings not only reaffirm age as the predominant risk factor for myopia but also illuminate the complex, non-linear nexus between economic development and visual health, alongside revealing a substantial “correction gap” within current prevention frameworks.

The dominant role of age—with an effect size (η^2^ = 0.1132) fivefold greater than that of city-level factors—aligns with prior epidemiological evidence (1, 4, 17, 18). Notably, this study identifies the 5–7-year age bracket as a critical window demarcating rapid refractive deterioration. This finding carries profound public health implications: myopia prevention mandates an anticipatory shift toward the preschool period, as conventional “post-enrollment screening” strategies risk forfeiting the optimal intervention window. Consequently, resource prioritization should pivot decisively toward this transition phase. This necessitates guaranteeing access to early refraction services and appropriate, updated correction, alongside optimizing the school visual environment (e.g., lighting, green space) to mitigate physiological deterioration. Future resource allocation should prioritize preschool and lower primary grades, emphasizing vigilant monitoring of hyperopic reserve depletion.

### 4.1 Implications of the “Xiamen Paradox” and the “Quanzhou Model”

A salient finding of this study is the stark divergence in behavioral responses across regions. The “Xiamen Paradox” manifests as superior refractive status fostered by economic affluence, yet concurrently cultivates a cognitive bias wherein “adequate unaided vision negates the need for correction,” resulting in an exceptionally high uncorrected rate (94%). Conversely, the “Quanzhou Model” illustrates “symptom-driven” behavior modification: the substantial myopia burden (high myopia rate: 19.0%) and the resultant subjective urgency of blurred vision compel greater adherence to correction (spectacle-wearing rate: 65.1% in senior high). This contrast underscores that economic advancement does not automatically translate to improved eye health and may, in fact, engender a “health illusion.” More broadly, these findings challenge the prevailing assumption that rising regional GDP will inherently translate into improved visual health and equitable access to quality eye care. Instead, achieving genuine visual health equity necessitates targeted investments in behavioral change rather than mere infrastructure expansion. At the individual level, enhancing correction coverage may hinge less on reducing spectacle costs and more critically on rectifying parental and student perceptions regarding the sequelae of mild refractive error. These patterns remained robust after adjusting for age and sex, underscoring that the observed behavioral divergences are not mere artifacts of demographic compositional differences between cities. Future interventions must therefore prioritize behavioral nudges in affluent cities, occupational health integration in industrial hubs, and equitable resource allocation in heterogeneous regions.

### 4.2 Substantial unmet needs and deficits in correction quality

The data reveal a sobering reality: despite residing in a robust economic region, only 22.6% of records wore spectacles among 856,274 valid screening records in the primary school group, leaving 77.4% uncorrected. Moreover, among existing spectacle wearers, only half achieved adequate correction. This signifies a dual rupture in the public health cascade from “screening” to “effective correction.” Quanzhou epitomizes this deficit: while exhibiting a high spectacle-wearing rate, its low adequate correction rate (41.6%) reveals that “possessing spectacles” does not equate to “seeing clearly.” This points to systemic shortcomings in refractive accuracy and timely prescription updates within Quanzhou, necessitating a strategic pivot from merely promoting “spectacle provision” toward ensuring “quality correction” and “regular follow-up.”

### 4.3 County-level heterogeneity and precision governance

This study demonstrates that the true disparity in myopia burden resides at the county level (intra-regional difference: 1.66 D) rather than solely at the prefectural level. This challenges conventional policymaking practices that rely on uniform strategies across entire cities. Such heterogeneity is particularly evident in Zhangzhou, which is characterized by starkly uneven development across its counties. For instance, the profound intra-city disparities within Zhangzhou necessitate a departure from “one-size-fits-all” mandates toward differentiated strategies formulated at the county or even community level, guided by local refractive baselines. Furthermore, the alarmingly high prevalence of anisometropia among senior high school students (32.1%) cautions against focusing solely on myopia prevalence (quantity); instead, attention must shift to binocular visual dysfunction (quality) to preempt amblyopia.

### 4.4 Generalizability and policy implications

The observed heterogeneity among the three cities mirrors three archetypal developmental paradigms within China's prosperous coastal regions. Xiamen epitomizes the “High-GDP-per-capita Developed City,” where superior healthcare resources coexist with a cognitive bias favoring “visibility over correction.” Quanzhou represents the “Industrial Hub City with High Total GDP and Dense Migrant Populations,” where intense industrial pressures drive high myopia burdens, yet pragmatic “pain-driven” behaviors foster higher adherence to correction. Conversely, Zhangzhou embodies the “Multi-tiered Socioeconomic City with Pronounced Urban-Rural Divides,” highlighting how fragmented governance sustains persistent disparities even within a single municipality. Recognizing these distinct profiles is paramount for precision public health policymaking; a monolithic approach is inadequate. Interventions must align with the specific socioeconomic DNA of each locale—emphasizing behavioral nudges in affluent cities, occupational health integration in industrial hubs, and equitable resource allocation in heterogeneous regions.

### 4.5 Limitations and future directions

Several limitations warrant consideration. First, the analytic unit was the screening encounter rather than the unique individual. However, we addressed this by performing sensitivity analyses at the student level and employing GEE with student-level clustering, which revealed a substantial intraclass correlation coefficient (ICC = 0.473) for repeated measures. Second, although GEE effectively corrected standard errors for clustering, fully specified multilevel mixed-effects models—particularly BMMLR—capable of jointly modeling multiple binary outcomes (visual impairment, uncorrected status, adequate correction) while accommodating school and county random effects were computationally infeasible at this scale (>4 million encounters, >1.6 million students). Future studies employing Bayesian multilevel approaches or regionally stratified subsamples may overcome this constraint. Third, as a school-based screening program, our dataset lacks individual-level contextual variables—such as parental socioeconomic status, household proximity to optical shops, or school-specific vision care policies—that could further elucidate the mechanisms underlying the “Xiamen Paradox” and “Quanzhou Model.” Consequently, causal interpretations are necessarily limited to ecological associations at the city and county levels. Future studies integrating administrative education records, household surveys, or optometric service mapping are warranted to disentangle these multifactorial pathways. Fourth, Quanzhou contributed a relatively smaller proportion of records (14.8%, *n* = 660,902) compared to Xiamen and Zhangzhou. While this imbalance may theoretically affect precision, the absolute sample size remains substantial—particularly within key subgroups. Crucially, sensitivity analyses employing GEE with student-level clustering, as well as analyses restricted to one record per student, yielded highly consistent effect sizes and prevalence estimates. These findings confirm that the imbalance does not materially compromise the robustness or comparability of city-level and age-stratified estimates. Fifth, the absence of cycloplegia represents a significant measurement limitation. The notably myopic SE values observed in preschoolers (e.g., −0.97 D in Zhangzhou) are physiologically implausible under cycloplegia and likely reflect accommodative bias inherent to non-cycloplegic autorefraction in young children. Such bias may lead to an overestimation of myopia and an underestimation of physiological hyperopia, particularly in the preschool and early primary school groups. Finally, formal amblyopia diagnosis and referral tracking were beyond our screening scope, which focused on refractive surveillance. As amblyopia requires clinical assessments unavailable in school settings, we could not ascertain its true burden or validate referral pathways, highlighting a direction for future program integration.

## 5 Conclusion

This large-scale, real-world evidence study, encompassing 4.47 million screening encounters across Southern Fujian, delineates a critical “correction gap” that belies the region's economic prosperity. We demonstrate that while economic advancement provides the infrastructural capacity for eye care, it does not automatically confer optimal visual health, as evidenced by the “Xiamen Paradox” and the substantial burden of high myopia in Quanzhou. Crucially, our findings reposition age—not economic status—as the predominant driver of refractive error, identifying the 5–7-year window as a pivotal period for public health intervention. The stark intra-regional disparities observed at the county level further challenge the utility of one-size-fits-all policies, advocating instead for precision governance tailored to local socioeconomic contexts. Moving forward, the focus must transition from mere screening volume to ensuring the quality and continuity of care, bridging the chasm between detection and effective correction. Ultimately, this study provides a transferable roadmap for transforming regional economic capital into tangible ocular health dividends, aligning with global efforts to eliminate preventable visual impairment among future generations.

## Data Availability

The raw data supporting the conclusions of this article will be made available by the authors, without undue reservation.

## Supplementary material

Supplementary Figure S1 STROBE-compliant participant flow diagram. The STROBE-compliant flow diagram detailing participant inclusion and exclusion, with denominators reported at each step.

## References

[1] Xu L Li H Li F Zhang T Yan J Yan H . Investigating the trajectories of poor vision in children and adolescents in Wuhan, China 2016 to 2019: prospective cohort study. JMIR Public Health Surveill. (2025) 11:e53028. doi: 10.2196/53028 39964957 PMC11855164

[2] Lin B Chen LL Li DK. Mendelian randomization analysis reveals a causal relationship between preterm birth and myopia risk. Front Pediatr. (2024) 12:1404184. doi: 10.3389/fped.2024.1404184 39091988 PMC11291316

[3] Liu H Li R Zhang Y Zhang K Yusufu M Liu Y . Economic evaluation of combined population-based screening for multiple blindness-causing eye diseases in China: a cost-effectiveness analysis. Lancet Glob Health. (2023) 11:e45665. doi: 10.1016/S2214-109X(22)00554-X 36702141

[4] Bao WW Zhao Y Dadvand P Jiang N Chen G Yang B. Urban greenspace and visual acuity in schoolchildren: a large prospective cohort study in China. Environ Int. (2024) 184:108423. doi: 10.1016/j.envint.2024.108423 38241831

[5] Wang Y Li C Zhang H Ma Y. The impact of vision care program on the mental health of migrant children in Eastern China: evidence from a cluster-randomized controlled trial. Int J Equity Health. (2026) 25. doi: 10.1186/s12939-026-02854-7 PMC13235173 42015257

[6] Oke I Slopen N Hunter DG Wu AC. Vision testing for adolescents in the US. JAMA Ophthalmol. (2023)141:1068–72. doi: 10.1001/jamaophthalmol.2023.4475 37824151 PMC10570910

[7] Kallem M Guo X Dai X Ambrosino C Nguyen A Friedman DS . Associations between School-Based Vision Program Outcomes and School Characteristics in 410 Schools. Ophthalmology. (2025) 132:452–60. doi: 10.1016/j.ophtha.2024.11.012 39542178

[8] Neitzel AJ Wolf B Guo X Shakarchi AF Madden NA Repka MX . Effect of a randomized interventional school-based vision program on academic performance of students in grades 3 to 7: a cluster randomized clinical trial. JAMA Ophthalmol. (2021) 139:1104–14. doi: 10.1001/jamaophthalmol.2021.3544 34499111 PMC8430909

[9] Zhang X Tang J Wang Y Yang W Wang X Zhang R . Visual environment in schools and student depressive symptoms: insights from a prospective study across multiple cities in eastern China. Environ Res. (2024) 258:119490. doi: 10.1016/j.envres.2024.119490 38925465

[10] Li R Zhang K Li SM Zhang Y Tian J Lu Z . Implementing a digital comprehensive myopia prevention and control strategy for children and adolescents in China: a cost-effectiveness analysis. Lancet Reg Health West Pac. (2023) 38:100837. doi: 10.1016/j.lanwpc.2023.100837 37520278 PMC10372367

[11] Liu M Wu X Li Z Tan D Huang C. Assessment of eye care apps for children and adolescents based on the mobile app rating scale: content analysis and quality assessment. JMIR Mhealth Uhealth. (2024) 12:e53805. doi: 10.2196/53805 39269760 PMC11437221

[12] Yang BY Li S Zou Z Markevych I Heinrich J Bloom MS . Greenness surrounding schools and visual impairment in Chinese children and adolescents. Environ Health Perspect. (2021) 129:107006. doi: 10.1289/EHP8429 34704791 PMC8549527

[13] Zhu XQ Sun Y Lin R Luo YN Li JX Zhang YD . School greenspace and myopia incidence and burden in Chinese children and adolescents: the Guangzhou children and adolescents cohort study. Environ Res. (2026) 296:124062. doi: 10.1016/j.envres.2026.124062 41720444

[14] Zhang C Wang C Guo X Xu H Qin Z Tao L. Effects of greenness on myopia risk and school-level myopia prevalence among high school-aged adolescents: cross-sectional study. JMIR Public Health Surveill. (2023) 9:e42694. doi: 10.2196/42694 36622746 PMC9871879

[15] Maedel S Evans JR Harrer-Seely A Findl O. Intraocular lens optic edge design for the prevention of posterior capsule opacification after cataract surgery. Cochrane Database Syst Rev. (2021) 8:Cd012516. doi: 10.1002/14651858.CD012516.pub2 34398965 PMC8406949

[16] Bellsmith KN Gale MJ Yang S Nguyen IB Prentiss CJ. Validation of home visual acuity tests for telehealth in the COVID-19 Era. JAMA Ophthalmol. (2022) 140:465–71. doi: 10.1001/jamaophthalmol.2022.0396 35357405 PMC8972145

[17] Kang MT Hu Y Wang N Fu J Zhou A Liu Y . Deep learning prediction of childhood myopia progression using fundus image and refraction data. JAMA Netw Open. (2026) 9:e2553543. doi: 10.1001/jamanetworkopen.2025.53543 41587032 PMC12836131

[18] Pan Z Xian H Li F Wang Z Li Z Huang Y . Myopia and high myopia trends in Chinese children and adolescents over 25 years: a nationwide study with projections to 2050. Lancet Reg Health West Pac. (2025) 59:101577. doi: 10.1016/j.lanwpc.2025.101577. 40568343 PMC12192684

